# Supplementary material for: Properties of Therapeutic Deep Eutectic Solvents of l-Arginine and Ethambutol for Tuberculosis Treatment
Source: Molecules. 2018 Dec 24;24(1):55. doi: 10.3390/molecules24010055 (PMC6337512; doi:10.3390/molecules24010055)
Supplement: Supplementary file 1 [file molecules-24-00055-s001.pdf]

Supplementary Materials

Properties of Therapeutic Deep Eutectic Solvents of L-Arginine and Ethambutol for Tuberculosis Treatment

Filipa Santos [1], Maria Inês P.S. Leitão [2] and Ana Rita C. Duarte [1,*]

[1] LAQV, REQUIMTE, Departamento de Química da Faculdade de Ciências e Tecnologia, Universidade Nova de Lisboa, 2829-516 Caparica, Portugal; mfca.santos@campus.fct.unl.pt
[2] ITQB–Instituto de Tecnologia Química e Biológica, Universidade Nova de lisboa, Estação Agronómica Nacional, Av. Da República, 2780-157 Oeiras, Portugal; inesleitao@itqb.unl.pt
* Correspondence: aduarte@fct.unl.pt; Tel.: +351-212-949-680

Table S1. Different eutectic systems prepared, during optimization process.

Component A	Component B	Component C	Molar Ratio	Visual Aspect
Citric Acid	L-Arginine		1:1; 1:2; 2:1	White Solid
Citric Acid	L-Arginine	H_2O	1:1:3; 1:2:3; 2:1:3	White Solid
Citric Acid	L-Arginine	H_2O	1:1:5; 1:2:5	Transparent viscous liquid with white precipitate
Citric Acid	Ethambutol		1:1; 1:2; 2:1; 5:2; 7:3	White Solid
Citric Acid	Ethambutol	H_2O	1:2:4; 2:1:6; 4:1:4; 4:1:5; 4:1:7; 4:1:9; 5:1:2; 5:1:4; 5:1:9	White Solid
Citric Acid	Ethambutol	H_2O	1:1:6	Transparent viscous liquid with white precipitate

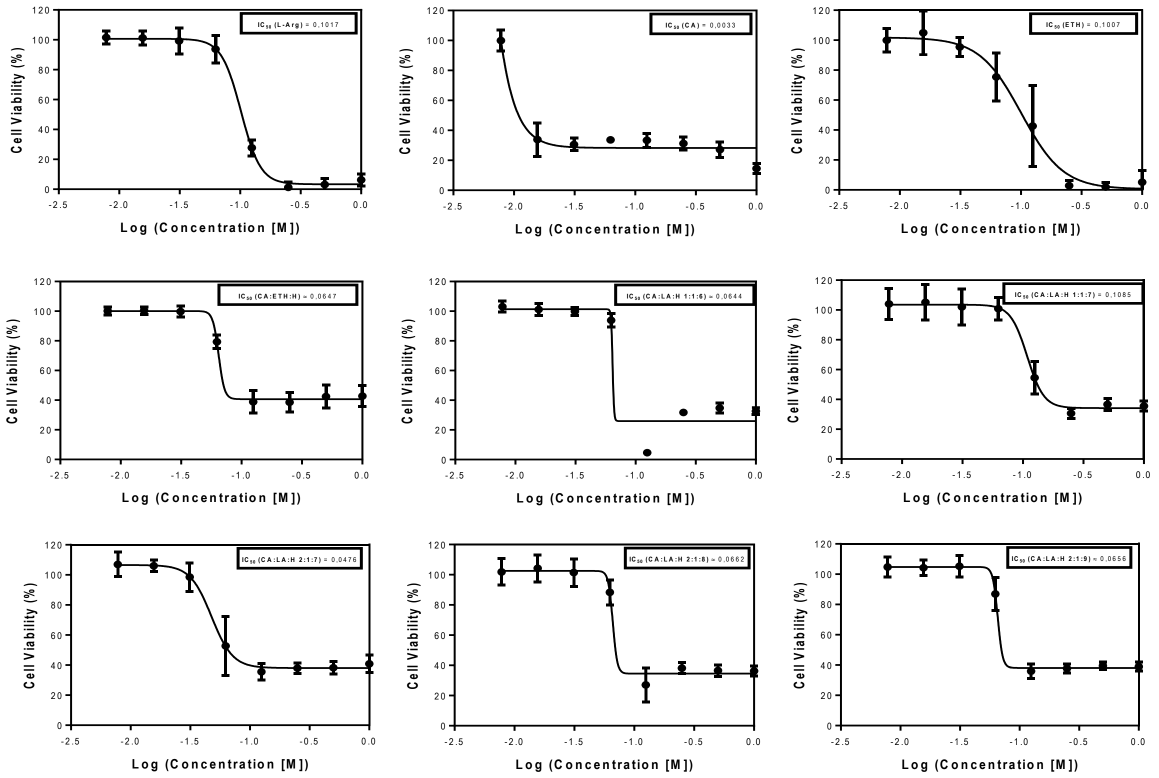

Figure S1. IC_{50} graphs with log concentrations and cell viability used to determine IC_{50}.

Table S2. ^{1}H and ^{13}C NMR chemical shifts.

Compound	Chemical Shift ^{1}H (ppm)						Chemical Shift ^{13}C (ppm)			
	**-CH₂**	**-CH₂**	**-COH**	**-COH**	**-OH**	**-NH**	**-C=O**	**2 x -C=O**	**-COH**	**2 x -CH₂**
Citric Acid	2,75	2,65	12,37	5,15	-	-	174,83	171,58	72,74	43,01
Ethambutol	-	-	-	-	9,24	5,40	-	-	-	-
Citric Acid:Ethambutol:H_2O (2:1:10)	2,75	2,65	12,34	5,15	9,14	5,40	174,67	171,45	72,59	42,82
Citric Acid:L-Arginine:H_2O (1:1:6)	2,57	2,50	-	-	-	-	177,34	172,09	71,87	44,50
Citric Acid:L-Arginine:H_2O (1:1:7)	2,58	2,51	-	-	-	-	176,97	171,70	71,67	44,29
Citric Acid:L-Arginine:H_2O (2:1:7)	2,64	2,56	-	-	-	-	176,12	171,52	71,99	43,72
Citric Acid:L-Arginine:H_2O (2:1:8)	2,66	2,57	-	-	-	-	176,02	171,65	72,14	43,66
Citric Acid:L-Arginine:H_2O (2:1:9)	2,65	2,56	-	-	-	-	176,28	171,82	72,22	43,82
